# High-flux hemodialysis combined with nutritional intervention: Effects on the nutritional status and clinical outcomes of patients with renal failure

**DOI:** 10.1371/journal.pone.0345943

**Published:** 2026-03-27

**Authors:** Yang Liu, Rongrong Shen, Xuehua Pu, Yaqing Zhou, Binbin Zhang

**Affiliations:** 1 Department of Clinical Nutrition, Haian Hospital of Traditional Chinese Medicine Affiliated to Nanjing University of Chinese Medicine, Nantong, Jiangsu Province, China; 2 Department of Critical Care Medicine, Jianhu People’s Hospital, Yancheng, Jiangsu Province, China; 3 The Affiliated Taizhou People’s Hospital of Nanjing Medical University, Taizhou, Jiangsu, China; 4 Nanjing Medical University, Nanjing, Jiangsu Province, China; 5 Department of Critical Care Medicine, Hai’an People’s Hospital, Nantong, Jiangsu Province, China; 6 Department of Blood Purification Center, Affiliated Hospital of Nantong University, Nantong, Jiangsu Province, China; Cairo University Kasr Alainy Faculty of Medicine, EGYPT

## Abstract

**Background:**

To evaluate the association between high-flux–based hemodialysis prescription combined with nutritional intervention and nutritional status and clinical outcomes in renal failure patients.

**Methods:**

Patients admitted between January 2020 and August 2024 were divided into an observation group (high-flux hemodialysis, n = 115) and a control group (Conventional blood purification, n = 115). Renal function [β2-microglobulin (β2-MG), serum creatinine (Scr), blood urea nitrogen (BUN)], inflammatory markers [interleukin-6 (IL-6), C-reactive protein (CRP), tumor necrosis factor-α (TNF-α)], and nutritional indices [serum albumin (ALB), prealbumin (PA), transferrin (SF)] were compared. Cox multivariate analysis identified prognostic factors.

**Results:**

Both groups exhibited post-treatment reductions in uremic markers (β2-MG: P < 0.05; Scr: P < 0.05; BUN: P < 0.05) and inflammation (IL-6: P < 0.05; CRP: P < 0.05; TNF-α:P < 0.05), with no baseline differences (P > 0.05). The observation group demonstrated significantly greater reductions in uremic markers and inflammatory markers versus controls (P < 0.05). Nutritional parameters (ALB, PA, SF) increased post-treatment in both groups (P < 0.05). Survival rates were higher in the observation group (P < 0.05). Deceased patients were predominantly≥60 years old, had cardiovascular comorbidities, and exhibited higher conventional blood purification utilization (P < 0.05).Lower ALB (P < 0.05) and elevated IL-6, CRP, TNF-α (P < 0.05) correlated with mortality.A reduced Cox model identified IL-6 (HR: 1.107, 95%CI: 1.063–1.153) and TNF-α (HR: 1.069, 95%CI: 1.012–1.130) as independent predictors of poor outcomes (P < 0.05).

**Conclusions:**

High-flux-based hemodialysis prescription with nutritional support was associated with more effective clearance of uremic markers, improvements in nutritional status and inflammatory response, as well as better survival in renal failure patients. Elevated IL-6 and TNF-α levels were identified as significant predictors of mortality in the reduced Cox model.

## 1 Introduction

Blood purification is considered an important treatment method for patients with renal failure as it facilitates an effective removal of metabolic wastes from the patient’s body and regulation of the electrolyte balance and acid-base balance in the patients [1,2]. Low-flux blood purification is an advanced blood purification technique used widely for patients as it is effective in the removal of small molecule toxins. However, the limited ability of this method to remove large molecule toxins prevents its widespread applicability. high-flux hemodialysis [3], on the other hand, has been demonstrated to enhance toxin removal. High-flux hemodialysis involves purification of the blood at a higher rate using a membrane or filter with greater permeability, a dialysis membrane with a large pore size, a high filtration rate, and a wide range of indications. While blood purification is an important treatment method for patients with renal failure, it affects the nutritional status of patients. Miao et al.[4] reported that malnutrition in patients with blood purification increases the risk of rehospitalization, death, and other factors indicating a poor prognosis, raising the requirement to assess patients regularly for their nutritional status throughout the treatment period and then providing the patients with nutritional interventions to improve the therapeutic efficacy and prognosis. Research on the effects of using different blood purification modalities in combination with nutritional interventions on the nutritional indicators and clinical outcomes of patients with renal failure is scarce. In addition, the individual characteristics of patients, the treatment modalities used, and the comorbidities in patients may affect the clinical outcomes of patients with renal failure and undergoing blood purification [5,6]. An early identification of risk factors would assist clinicians in formulating individualized countermeasures. This single-center, retrospective study therefore aimed to compare the effects of high-flux-based hemodialysis prescription versus conventional hemodialysis, both combined with a structured nutritional intervention, on renal function, inflammatory markers, nutritional status, and survival in patients with end-stage renal disease initiating renal replacement therapy. This study was conducted in the hemodialysis center and focuses on patients with end-stage renal disease undergoing maintenance hemodialysis.The objective was to provide a scientific basis for the treatment of renal failure.

## 2. Study subject and methods

### 2.1. Study subjects

Patients with end-stage renal disease initiating renal replacement therapy (RRT) who were admitted to Hai’an People’s Hospital between January 2020 and August 2024 for maintenance hemodialysis were screened for this study. This study specifically focused on patients newly initiating renal RRT. During the study period, a total of 290 patients commenced RRT and were assessed for eligibility. Of these, 60 patients were excluded for the following reasons: combination of malignant tumors (n = 15), presence of active systemic autoimmune or infectious diseases (n = 12), severe dysfunction of major organs such as heart, liver, or lungs (n = 10), pregnancy or lactation (n = 2), history of severe psychiatric disorders or known allergies to dialysis materials (n = 6), switching to peritoneal dialysis or receiving kidney transplantation during the initial treatment phase (n = 11), and incomplete clinical data or immediate loss to follow-up (n = 4). Consequently, 230 eligible patients were ultimately enrolled and constituted the study cohort.The inclusion criteria were as follows: (1) patients who met the indications of blood purification for renal failure [7]; (2) the duration of standardized blood purification was ≥ 6 months; (3) complete clinical data were available. The exclusion criteria were: (1) the presence of a combination of malignant tumors, immune system diseases, and acute and chronic infections; (2) dysfunction of the heart, brain, liver, and any other organ; (3) pregnant or lactating females; (4) psychiatric patients and patients with allergies; (5) switching to peritoneal dialysis during treatment. The included patients were divided into two groups based on the treatment option used: observation group (high flux blood purification) and control group (conventional blood purification). Each group included 115 patients. The observation group had 73 males and 42 females, with 39 patients of age ≥ 60 years and 74 patients of age < 60 years. The average disease duration in the observation group was 6.41 ± 2.26 years. The control group had 78 males and 37 females, with 49 patients having age ≥ 60 years and 66 cases having age < 60 years. The average disease duration in the control group was 6.36 ± 2.01 years. The two groups did not differ in terms of general information, such as gender and age (P > 0.05). The study was conducted strictly in accordance with the principles of the Declaration of Helsinki.The study was approved by Medical Ethics Committee of Hai’an People’s Hospital (HKL2024023) and the Ethics Committee considered it a retrospective study and waived informed consent.Data were accessed for research purposes on September 20, 2024. The authors had no access to information that could identify individual participants during or after data collection.

### 2.2. Methodology

All enrolled patients received the standard care routinely provided at our hospital’s Hemodialysis Center for patients commencing maintenance hemodialysis. This care included symptomatic management to regulate blood pressure, blood glucose, and maintain water-electrolyte and acid-base balance. Additionally, a standardized yet individualized nutritional intervention was implemented to address common issues such as anorexia and dialytic nutrient losses. This nutritional strategy began with a comprehensive assessment by a clinical dietitian, using the Patient-Generated Subjective Global Assessment, biochemical parameters, and anthropometric measurements. Subsequently, a personalized dietary prescription was formulated, specifying daily targets for energy (138.07–146.66 kJ·kg ⁻ ¹·d ⁻ ¹), protein (1.2–1.4g·kg ⁻ ¹·d ⁻ ¹), and other nutrients. Practical implementation support was provided through educational sessions on food selection, written materials with sample menus, and management strategies such as small, frequent meals. Compliance was monitored through a multi-faceted approach. This included bi-weekly 3-day dietary diaries reviewed by the dietitian, monthly biochemical monitoring of nutritional markers, and regular follow-up interviews every 4–6 weeks to offer support and adjust the plans.

According to the group assignment, the observation group underwent high-flux hemodialysis. An FX60 dialyzer (Fresenius, Germany) was used, which had an ultrafiltration coefficient of 46 mL·h ⁻ ¹·mmHg ⁻ ¹, a membrane area of 1.6 m², a dialysate flow rate of 500 mL/min, and a blood flow rate ranging from 300 to 360 mL/min. Meanwhile, the control group received conventional hemodialysis following the standard unit protocol. An AK96 hemodialyzer (Baxter) was employed, with an ultrafiltration coefficient of 5.5 mL·h ⁻ ¹·mmHg ⁻ ¹, a membrane area of 1.0 m², a dialysate flow rate of 500 mL/min, and a blood flow rate of 200–260 mL/min. Both groups used upper limb arteriovenous fistula or deep vein cannulation and low molecular heparin anticoagulation. The hemodialysis sessions lasted for 4 hours and were carried out three times a week.

### 2.3. Observation indicators

Venous blood samples were collected under standardized conditions. Baseline samples were obtained after an overnight fast, immediately prior to the initial hemodialysis session. Post-treatment samples were collected 24 hours after completion of the final hemodialysis session at the 6-month post-enrollment time point to minimize acute fluctuations associated with a single dialysis procedure.

(1) Uremic markers and inflammation. Uremic markers included β2-microglobulin (β2-MG), serum creatinine (Scr), and blood urea nitrogen (BUN), which reflect the accumulation of metabolic wastes. Inflammatory factors included interleukin-6 (IL-6), C-reactive protein (CRP), and tumor necrosis factor-α (TNF-α). (2) Nutritional indicators. Serum albumin (ALB), prealbumin (PA), and transferrin (SF) in the two groups prior to and after treatment were compared. (3) The clinical outcomes were compared by analyzing the follow-up records (containing data on survival and death) of the patients in the two groups.

### 2.4. Statistical methods

SPSS 20.0 software was employed to conduct the statistical analyses. Count data were expressed as rates (%) and analyzed using the *x* ^2^ test. The continuous data conforming to normality were expressed as mean ± standard deviation (± *s*), and the two groups of data were compared using the t-sample test. Patient survival in the two groups was determined based on the KM survival curves, and the factors affecting the clinical outcomes were determined using the Cox multifactorial regression analysis. The differences with P < 0.05 were considered statistically significant.

Consistency verification for Cox regression included: ①Outcome definition: death = 1, survival = 0; ②Categorical variable coding: age (≥60 years = 1/ < 60 years = 0), cardiovascular comorbidity (yes = 1/no = 0), treatment modality (high-flux = 1/conventional = 0); ③ Double data entry and SPSS 20.0 validation to exclude errors. Candidate variables for the multivariate Cox model were selected based on clinical relevance and univariate analysis results (P < 0.10). The event-per-variable ratio met the requirement of ≥5 for the reduced model, ensuring statistical robustness.

### Model stability and sensitivity analyses

Given the limited number of endpoint events (n = 25), we implemented multiple strategies to mitigate overfitting risk and verify model robustness: ① Variable selection: Candidate variables were screened based on clinical relevance and univariate analysis results (P < 0.10) to avoid redundant covariates; ② Model simplification: A reduced model retaining only IL-6 and TNF-α was prioritized as the primary analysis. The event-per-variable (EPV) ratio was 12.5 for the reduced model versus 3.6 for the full model; ③ Sensitivity analyses: We performed bootstrap resampling to evaluate the consistency of HR estimates and leave-one-out cross-validation to assess the predictive performance of the reduced model; ④ Proportional hazards assumption: Verified via Schoenfeld residual plots and the global test.

## 3. Results

### 3.1. Renal function and inflammation

Prior to treatment, the Uremic markers (β2-MG, Scr, BUN) and inflammation (IL-6, CRP, TNF-α) indices did not differ between the two groups (*P* > 0.05).Uremic markers and inflammation were significantly lower after 6 months of treatment compared to baseline in both groups (*P* < 0.05), indicating effective toxin clearance and mitigation of inflammatory response. After treatment, the observation group showed significantly lower levels of uremic markers and inflammatory factors than the control group (*P* < 0.05), suggesting superior dialysis adequacy and anti-inflammatory effects of the high-flux–based hemodialysis prescription.Detailed data are provided in Table 1.

**Table 1:** Comparison of uremic markers and inflammatory indices between the two groups of patients (± *s*).

groups		Observation group (n = 115)	Control group (n = 115)	t-value	*P*-value (Intragroup)	*P*-value (Intergroup, Post-treatment)
β2-MG (mg/L)	pre-treatment	6.41 ± 0.79	6.24 ± 0.79	1.687		0.093
	post-treatment	1.86 ± 0.28^#^	2.88 ± 0.53^#^	18.579	<0.001	<0.001
Scr (μmol/L)	pre-treatment	736.64 ± 72.28	737.04 ± 81.93	0.039		0.969
	post-treatment	421.27 ± 21.08^#^	476.30 ± 23.48^#^	18.706	<0.001	<0.001
BUN (mmol/L)	pre-treatment	31.80 ± 3.05	31.86 ± 3.21	0.126		0.900
	post-treatment	8.56 ± 0.27^#^	12.45 ± 0.63^#^	60.601	<0.001	<0.001
IL-6 (ng/L)	pre-treatment	189.04 ± 21.95	192.21 ± 26.92	0.980		0.328
	post-treatment	129.16 ± 12.70^#^	149.60 ± 15.63^#^	10.886	<0.001	<0.001
CRP (mg/L)	pre-treatment	11.70 ± 4.24	12.09 ± 5.00	0.627		0.531
	post-treatment	5.48 ± 1.61^#^	8.60 ± 1.50^#^	15.216	<0.001	<0.001
TNF-α (ng/L)	pre-treatment	43.14 ± 7.60	44.46 ± 9.30	1.185		0.237
	post-treatment	12.23 ± 1.60^#^	25.87 ± 2.20^#^	53.661	<0.001	<0.001
ALB (g/L)	pre-treatment	23.11 ± 2.69	23.10 ± 2.64	0.022		0.866
	post-treatment	39.60 ± 4.01^#^	40.25 ± 3.92^#^	1.236	0.218	0.218
PA (g/L)	pre-treatment	272.53 ± 21.97	270.07 ± 19.55	0.897		0.371
	post-treatment	290.22 ± 30.96^#^	286.22 ± 32.02^#^	0.964	0.336	0.336
SF (g/L)	pre-treatment	1.39 ± 0.24	1.43 ± 0.24	1.096		0.109
	post-treatment	1.68 ± 0.38^#^	1.68 ± 0.34^#^	0.274	0.913	0.913

Note:^#^ Intragroup comparison

*t*_（Observation）_ =57.396,43.466,82.264,24.475,14.908;33.655, 5.750, 6.707,

*P*_（Observation）_=0.000,0.000,0.000,0.000,0.000,0.000；0.000, 0.000, 0.000,

*t*_（Control）_ =41.403,32.371,66.031,14.839,7.163,20.812, 36.039, 4.293, 6.383,

*P*_（Control）_ = 0.000, 0.000, 0.000, 0.000, 0.000, 0.000, 0.000, 0.000, 0.000.

### 3.2. Nutritional indices

The nutritional indices ALB, PA, and SF in the two groups of patients after treatment were higher than the corresponding values noted prior to treatment (P < 0.05), although no significant differences were observed between the two groups after treatment (Table 1).

### 3.3. Clinical outcomes

**Fig 1:**
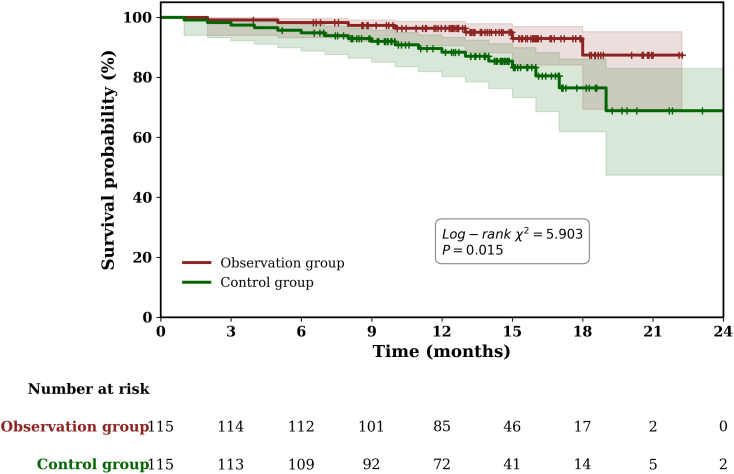
Kaplan-Meier survival curve.

On the follow-up conducted in August 2024, the following data were obtained: observation group follow-up duration, 14.58 ± 4.06 months; control group follow-up duration 13.55 ± 4.36 months; observation group death cases, 7 (6.09%, 7/115); control group death cases, 18 (15.65%, 18/115). No patients were lost to follow-up during the study period, and all censoring was attributable to patients remaining alive at the end of follow-up. Survival rate in the observation group was greater than that in the control group (*x* ^2^ = 5.903, P = 0.015). Fig 1 provides detailed data.

### 3.4. Factors influencing the clinical outcomes

Twenty-five of the 230 patients with renal failure died, while 205 patients survived. The ratio of age ≥ 60 years, comorbid cardiovascular disease, and conventional blood purification treatment method were the factors that were higher among the patients who died (P < 0.05. The ALB levels were lower among patients who died than among those who survived (*P* < 0.05). The levels of IL-6, CRP, and TNF-α were higher among patients who died than among those who survived (*P* < 0.05). Detailed data are provided in Table 2.

**Table 2:** Comparison of the basic data of patients with different clinical outcomes of renal failure.

Indicators	Death group (n = 25)	Survival group (n = 205)	statistical value	P-value
Gender (cases)			0.069	0.793
male	17 (68.00)	134 (65.37)		
women	8 (32.00)	71 (34.63)		
Age (cases)			40.480	<0.001
≥ 60 years	23 (92.00)	57 (27.80)		
<60 years	2 (8.00)	148 (72.20)		
Duration of illness (years)	6.19 ± 2.00	6.41 ± 2.15	0.492	0.623
BMI (kg/m2)	23.16 ± 2.02	23.72 ± 2.03	1.301	0.194
Smoking (cases)			0.053	0.818
Yes	7 (28.00)	53 (25.85)		
No	18 (72.00)	152 (74.15)		
Alcohol abuse (cases)			0.031	0.860
Yes	6 (24.00)	46 (22.44)		
No	19 (76.00)	159 (77.56)		
Combined gout (cases)			0.015	0.902
Yes	2 (8.00)	15 (7.32)		
No	23 (92.00)	190 (92.68)		
Combined cardiovascular disease (cases)			18.695	<0.001
Yes	17 (68.00)	53 (25.85)		
No	8 (32.00)	152 (74.15)		
Treatment (cases)			5.430	0.020
High-flux hemodialysis	7 (28.00)	108 (52.68)		
Conventional blood purification	18 (72.00)	97 (47.32)		
HbA1c (%)	5.81 ± 1.95	6.04 ± 1.87	0.561	0.575
β2-MG (mg/L)	6.53 ± 0.69	6.30 ± 0.80	1.349	0.179
PA (g/L)	271.34 ± 20.83	271.9 ± 20.83	0.009	0.993
SF (g/L)	1.43 ± 0.26	1.41 ± 0.24	0.367	0.714
Total cholesterol (mmol/L)	170.96 ± 25.65	174.53 ± 26.07	0.649	0.517
Triacylglycerol (mmol/L)	114.18 ± 20.07	115.27 ± 19.84	0.258	0.797
Hemoglobin (g/L)	103.28 ± 9.22	102.19 ± 9.64	0.534	0.594
Fasting blood glucose (mmol/L)	7.19 ± 0.78	7.10 ± 0.95	0.465	0.642
ALB (g/L)	21.96 ± 2.19	23.24 ± 2.68	2.309	0.022
Scr (μmol/L)	742.33 ± 76.59	736.17 ± 77.31	0.377	0.707
BUN (mmol/L)	31.09 ± 3.02	31.92 ± 3.13	1.260	0.209
IL-6 (ng/L)	238.46 ± 12.90	184.79 ± 18.51	14.073	<0.001
CRP (mg/L)	20.46 ± 4.97	10.85 ± 3.33	12.814	<0.001
TNF-α (ng/L)	58.29 ± 10.34	42.03 ± 6.29	11.236	<0.001

In light of the limited number of mortality events (n = 25), we prioritized the reduced Cox model (containing only IL-6 and TNF-α) as the primary analysis, given its optimal EPV ratio (12.5) and clinical relevance to the malnutrition-inflammation-atherosclerosis (MIA) syndrome in ESRD. For completeness and transparency, Table 3 presents the full multivariable model including all candidate variables, but the HRs of non-core variables should be interpreted with caution due to the full model’s low EPV ratio (3.6). Notably, the treatment modality showed a protective effect in the full model (HR = 0.330, 95%CI: 0.120–0.910, P = 0.032), consistent with the higher survival rate in the observation group.In contrast, age ≥ 60 years (HR = 2.084, 95%CI: 0.340–12.762) and comorbid cardiovascular disease (HR = 1.672, 95%CI: 0.650–12.490) showed trends toward increased mortality risk, which is consistent with the higher prevalence of these factors in the deceased group (Table 2). However, their statistical insignificance in the multivariate model may be attributed to collinearity with inflammatory markers (IL-6 and TNF-α) and the limited number of endpoint events.

**Table 3:** Deaths due to renal failure – Cox multifactorial regression analysis.

	variant	β	S.E.	Wald	P-value	HR (95% CI)
Full Model	Age	0.734	0.923	0.633	0.426	2.084 (0.340-12.762)
	Combined cardiovascular disease	0.514	0.481	1.143	0.285	1.672 (0.650-12.490)
	Treatment	−1.116	0.519	4.620	0.032	0.330 (0.120-0.910)
	ALB	−0.131	0.108	1.487	0.223	0.877 (0.710-1.083)
	CRP	0.025	0.039	0.408	0.523	1.025 (0.950-1.106)
Reduced Model	IL-6	0.102	0.021	23.859	<0.001	1.107 (1.063-1.153)
	TNF-α	0.067	0.028	5.595	0.018	1.069 (1.012-1.130)

Note:In the Cox proportional hazards model, the outcome event was death. The reference groups for categorical variables were as follows: age < 60 years, absence of cardiovascular disease, and receipt of conventional hemodialysis. An HR > 1 indicates an increased risk of mortality associated with the listed category relative to its reference.The multivariate Cox model was constructed with awareness of the limited number of events. The presented model includes variables significant in univariate analysis and of clinical importance. The reduced model is the primary analysis due to better statistical stability. Reference groups for categorical variables: Age (<60 years), Combined cardiovascular disease (No), Treatment (conventional blood purification).

Sensitivity analysis confirmed the robustness of the primary findings: Bootstrap resampling(1,000 replicates) showed narrow 95% CIs for IL-6 (1.061–1.155) and TNF-α (1.011–1.132), with no significant fluctuations in HR estimates; leave-one-out cross-validation further validated the reduced model’s consistent predictive performance. These results confirm that IL-6 and TNF-α are stable independent predictors of mortality in patients with renal failure in the statistically robust reduced Cox model, with conventional risk factors not included due to event-per-variable constraints.

It is noteworthy that advanced age and comorbid cardiovascular disease, which were significant in the univariate analysis (Table 2), were not retained as independent predictors in the multivariate Cox model. This statistical phenomenon can be attributed to potential collinearity, as the physiological effects of aging and cardiovascular disease are closely associated with elevated levels of systemic inflammatory markers such as IL-6 and TNF-α. When these strongly correlated variables are included simultaneously, the model allocates the predictive power to the most direct and potent factors—in this case, the inflammatory cytokines. Additionally, the limited number of endpoint events (25 deaths) may constrain the statistical power to identify all independent risk factors reliably.Correlation analysis confirmed that age and comorbid cardiovascular disease were positively correlated with IL-6 (r = 0.31, P < 0.05) and TNF-α (r = 0.28, P < 0.05), which explained why they were not retained as independent predictors in the multivariate model. This further supports that inflammatory factors are more direct drivers of mortality in patients with renal failure, as identified in our primary reduced Cox model.

## 4. Discussion

This study demonstrates that high-flux hemodialysis, when combined with standardized nutritional support, is associated with superior clearance of uremic solutes, more effective mitigation of systemic inflammation, and a significant survival advantage compared to conventional hemodialysis in patients with ESRD. Crucially, findings from our reduced Cox model validate serum IL-6 and TNF-α levels as potent predictors of mortality, thereby highlighting the central role of the inflammatory response in determining clinical outcomes.The observed enhancement in the removal of uremic toxins and inflammatory mediators with high-flux hemodialysis is consistent with the known biophysical properties of high-permeability membranes [3,8,9].The reductions in Scr and BUN observed in this study do not indicate improvement in intrinsic renal function, as end-stage renal disease is characterized by irreversible loss of renal parenchymal function. Instead, these changes reflect the efficiency of hemodialysis in removing uremic toxins, which is consistent with the core function of renal replacement therapy. Our results provide clinical corroboration for the mechanistic premise that high-flux-based hemodialysis prescription offers a broader spectrum of solute clearance, a property linked to improved patient outcomes in prior study [10]. The significant attenuation of IL-6 and TNF-α in the high-flux group is of particular importance.

The 2024 KDIGO Clinical Practice Guideline for the Evaluation and Management of CKD underscores the pivotal role of chronic inflammation in the progression of kidney disease and the development of its complications [11].The quest to optimize hemodialysis efficacy, particularly through high-flux membranes, has been a focus of major international trials. The landmark HEMO study demonstrated that high-flux dialysis reduced the risk of specific causes of mortality, particularly in patients dialyzing for longer durations, highlighting the potential importance of removing larger solutes [10]. Subsequent studies like the MPO trial reinforced that high-flux membranes may confer a survival advantage, especially in high-risk subgroups such as those with hypoalbuminemia [12]. The ESHOL study further underscored the survival benefit of convective therapies, which share the high-permeability principle with high-flux hemodialysis, in removing middle molecules and inflammatory mediators. Against this backdrop, and in line with the recent emphasis in the 2024 KDIGO Clinical Practice Guideline for CKD on managing inflammation and complications [11], as well as the 2020 KDOQI Nutrition Guideline’s focus on individualized nutritional care [13], our study aimed to evaluate the combined role of high-flux hemodialysis and structured nutrition in a real-world, single-center cohort. Our data, which directly link the reduction of these specific cytokines with a survival benefit, offer empirical support for this strategic focus. This is highly consistent with the established pathophysiology of the malnutrition-inflammation-atherosclerosis (MIA) syndrome, in which cytokines like IL-6 and TNF-α are recognized as core mediators driving morbidity and mortality [14,15].

A key and instructive finding was the comparable improvement in nutritional parameters across both treatment groups. This outcome strongly implies that the rigorous, protocol-driven nutritional intervention was the principal driver of nutritional recovery, effectively offsetting the catabolic stress of dialysis therapy itself [16, 17]. This observation aligns with the emphasis placed by the KDIGO guideline on the necessity of individualized nutritional care as a foundational component of CKD management [11] and is supported by the conclusions of other major guidelines and studies [13]. The fact that both groups achieved similar nutritional status despite differences in dialysis efficacy suggests that the survival advantage in the high-flux group is more directly attributable to its superior control of uremia and inflammation [10] than to differential nutritional gains. This distinction reinforces the concept that managing the inflammatory milieu is a complementary and essential pathway to improving survival in a patient population where malnutrition and inflammation are synergistic drivers of poor outcomes [15].

Our findings regarding the superior efficacy of high-flux hemodialysis in clearing uremic markers and reducing inflammation are consistent with and extend the observations made by Luo Qiuju et al [18], who also reported beneficial effects on microinflammatory responses. After treatment, it was observed that the renal function and inflammation indices in the observation group were significantly lower than those in the control group (P < 0.05). This superior efficacy is consistent with the greater permeability and enhanced clearance capacity of high-flux membranes [8,9]. In addition, high-flux hemodialysis accomplishes a greater level of removal in a shorter duration, thereby reducing the number of dialysis sessions required and the risk of developing complications.

Further, the nutritional indices of patients in both groups after treatment were significantly higher than the corresponding values noted prior to treatment (P < 0.05), indicating that different blood purification modalities combined with nutritional interventions could alter the nutritional status of the patients with renal failure. The lack of a significant intergroup difference in nutritional parameters, despite their intragroup improvement, warrants careful interpretation. It strongly suggests that the structured nutritional intervention provided to all patients was the predominant factor driving the observed nutritional amelioration. Furthermore, this finding can be explained by the countervailing physiological effects of high-flux hemodialysis. On one hand, it may offer superior clearance of uremic toxins that contribute to anorexia and catabolism. On the other hand, it is also known to increase the convective loss of nutrients, including amino acids and vitamins [14,19]. Thus, the net nutritional outcome seen in the observation group may result from a balance between these positive and negative effects, ultimately appearing similar to the control group. This interpretation underscores that the applied nutritional support was crucial in both arms, potentially compensating for the higher nutrient losses associated with high-flux dialysis.

It is well recognized that hemodialysis itself can induce nutrient losses through multiple pathways, including dialysate losses of proteins and amino acids, anorexia related to uremia, and dietary restrictions [20]. The structured nutritional intervention employed in this study was specifically designed to counteract these challenges. The provision of individualized, high-protein dietary prescriptions aimed to compensate for dialytic losses, particularly crucial in the high-flux group. The educational and practical support directly addressed issues of anorexia and early satiety. Furthermore, the multi-faceted compliance monitoring system was critical for engaging patients in their own care outside the clinical setting, providing ongoing support, and allowing for timely adjustments to the nutritional plan, thereby promoting sustained adherence and improving the overall effectiveness of the intervention.In addition, imbalances in the metabolism of electrolytes, such as potassium, calcium, and phosphorus, and trace elements such as zinc and selenium during the treatment process may lead to nutrient loss [19, 21]. However, in the present study, no difference was noted in the nutritional indices between the two groups (P > 0.05), suggesting that nutritional intervention should be emphasized in patients with renal failure and undergoing blood purification treatment. In terms of clinical outcomes, the survival rate noted in the observation group was higher than that noted in the control group (x2 = 5.903, P = 0.015), suggesting a positive impact on the survival rate of patients in the observation group. The divergence between nutritional and survival outcomes offers critical insight. While both groups achieved comparable and significant improvements in key nutritional parameters (ALB, PA, SF), the high-flux hemodialysis group demonstrated a significantly higher survival rate. This dissociation strongly suggests that the survival benefit associated with high-flux hemodialysis is not primarily mediated through differential nutritional improvement, but rather through its superior efficacy in mitigating systemic inflammation, as evidenced by the significantly greater reductions in IL-6, CRP, and TNF-α levels in the observation group. These findings underscore the paramount importance of controlling the inflammatory milieu in ESRD, which appears to be a more direct driver of mortality risk than nutritional status per se in this cohort receiving standardized nutritional support. They align with and extend the concept of the malnutrition-inflammation-atherosclerosis (MIA) syndrome, highlighting that targeted reduction of pro-inflammatory cytokines may yield significant survival advantages even when nutritional parameters are stabilized.The baseline data and the laboratory indicators of patients with different clinical solutions were analyzed next to obtain a further comprehensive understanding of the prognostic factors influencing the prognosis of patients with renal failure and undergoing blood purification. The results revealed that age, comorbid cardiovascular disease, treatment modality, and the levels of ALB, IL-6, CRP, and TNF-α differed significantly between the patients who survived and those who died (P < 0.05). The results of Cox multifactorial regression analysis revealed IL-6 [HR (95% CI): 1.107 (1.063 ~ 1.153)] and TNF-α [HR (95% CI): 1.069 (1.012 ~ 1.130)] as risk factors affecting the clinical outcomes of patients with renal failure (P < 0.05). This finding indicates that, in patients with renal failure, each unit increase in IL-6 was associated with an approximately 10.7% higher risk of adverse clinical outcomes, and each unit increase in TNF-α with a 6.9% higher risk. Our results from the reduced Cox model confirm that IL-6 and TNF-α are strong predictors of mortality in this cohort. These findings suggest that inflammatory burden may serve as a more direct and potent contributor to poor clinical outcomes in this population.Accordingly, it is suggested that IL-6 and TNF-α may be used as potential biomarkers in the clinical management of patients with renal failure, and monitoring the levels of these two might play a key role in predicting patient prognosis and then developing individualized treatment plans for patients. In the present study, the effect of using high-flux hemodialysis combined with nutritional intervention for treating patients with renal failure was analyzed, and survival analysis was performed to provide a reference for patient prognosis. However, the study does have several limitations. Firstly, from the existing records, we were unable to retrieve and analyze the specific causes of death of the deceased patients. This information would have been highly valuable for further elucidating the potential mechanisms underlying the survival benefit observed in the high-flux group.Future studies with cause-specific mortality data are needed to determine if the survival benefit of high-flux hemodialysis is primarily driven by a reduction in cardiovascular or infection-related deaths. Secondly, the follow-up duration in this study was relatively short. With a mean follow-up period of around 14 months, it was not feasible to perform a comprehensive assessment of long-term survival and cardiovascular outcomes. Moreover, the alterations in the 5-year survival rate of patients following the use of different purification modalities in combination with nutritional intervention were not recorded. Additionally, no comparison was made with hemodialysis filtration and other therapeutic modalities, which could be a future research direction.Third, A potential limitation is the limited number of mortality events (n = 25), which constrains the statistical power of the full multivariable Cox model. Although we present the full model for transparency, its low EPV ratio (3.6) may lead to unstable HR estimates for non-core variables. To address this concern, we prioritized a reduced model focusing on IL-6 and TNF-α—two well-established core mediators of the MIA syndrome in ESRD. Multiple sensitivity analyses consistently confirmed that IL-6 and TNF-α remained strong predictors in the reduced model with minimal HR fluctuations, effectively mitigating concerns about model instability. Future prospective studies with larger sample sizes and more endpoint events are needed to validate the prognostic value of other candidate variables.the statistical power of the multivariable Cox regression analysis is constrained by the relatively low number of observed endpoint events (deaths, n = 25) relative to the number of covariates initially considered. This increases the risk of model overfitting and may affect the stability and precision of the hazard ratio estimates. Therefore, the results should be interpreted with a focus on the direction and strength of the associations (particularly for IL-6 and TNF-α) rather than the exact point estimates.

In summary, this single-center retrospective study suggests an association between the high-flux-based hemodialysis prescription and more significant reductions in uremic markers and inflammatory markers, as well as a higher short-term survival rate, compared to conventional hemodialysis in patients with renal failure, within the context of standardized nutritional support. The comparable improvement in nutritional indices among groups highlights the crucial role of concurrent nutritional intervention. Moreover, IL-6 and TNF-α were identified as independent risk factors for mortality in the statistically robust reduced Cox model. Nevertheless, due to the retrospective design, single-center nature, and relatively short follow-up period, causal relationships cannot be established, and the findings regarding long-term outcomes are restricted. Future prospective, multi-center studies with an extended follow-up are necessary to validate these findings and further clarify the underlying mechanisms.

## Supporting information

S1 Raw data Raw data for Fig 1. (TIFF)
